# The effects of illness perceptions on their medication attitudes among patients with schizophrenia

**DOI:** 10.1192/j.eurpsy.2021.1049

**Published:** 2021-08-13

**Authors:** J.-Y. Syu, E.C.-L. Lin

**Affiliations:** 1 Nursing, Chiayi Branch, Taichung Veterans General Hospital, Chiayi, Taiwan; 2 Nursing, National Cheng Kung University, Tainan, Taiwan

**Keywords:** illness perceptions, medication attitudes, schizophrénia

## Abstract

**Introduction:**

Antipsychotics are the primary treatment for patients with schizophrenia. However, medication non- adherence rate of schizophrenia patients is high. Illness perceptions have been identified as critical indicators to influence patients’ medication adherence and treatment process. Knowledge remains unclear about the effects of illness perceptions on medication attitudes among patients with schizophrenia.

**Objectives:**

This study aimed to investigate the effects of illness perceptions on medication attitudes among patients with schizophrenia.

**Methods:**

This cross-sectional study was conducted in a regional teaching hospital in southern Taiwan with a convenience sample of 200 patients with schizophrenia recruited. Two self-reported scales, Illness Perception Questionnaire-Revised (IPQ - R) and Drug Attitude Index - 10 (DAI - 10), were used to assess patients’ illness perceptions and medication attitudes. Positive illness perceptions mean patients believe their illness acute, noncyclical, fewer consequences and emotional representation. And have more personal control, treatment control, and illness coherence.

**Results:**

Patients’ illness perceptions were negative, with a little illness identity. Most of them believed that illness is more chronic and cyclical, causing negative consequences, lower self-control, and negative emotional expression. However, they thought treatment is moderately helpful for illness control, and the treatment effect is moderate. Multiple regression analysis showed that positive illness perceptions and negatively emotional representation could predict better medication attitudes.

**Conclusions:**

Our findings suggest that psychiatric mental health professionals could assess the illness perceptions of schizophrenia patients to influence their medication attitudes. Moreover, developing evidence-based interventions to improve their positive illness perceptions and decrease negative illness perceptions is needed.

